# 

**Published:** 1916-05-27

**Authors:** 


### Matrons' and Sisters' Appointments.

Arnold Military V.A.D. Hospital, Doncaster.—Miss C. S. Marshall has been appointed sister. She has been matron of Bridport Cottage Hospital and night superintendent at Southwark Infirmary. Miss Marshall was trained at Oldham General Hospital.

Tredegar Park Cottage Hospital (Mon.).—Miss Gertrude Thomas has been appointed matron. She was trained at the Royal Gwent Hospital, Newport, held various posts at Bolingbroke Hospital, Wandsworth Common, and later was assistant matron at Merthyr General Hospital and sister at Cambridge Research Hospital.

**NOTE.—Particulars of Appointments for insertion in this column will be welcomed.**

### Passing Events and Topics.

### The College of Nursing.

The Memorandum and Articles of Association of the College of Nursing, Limited, have been placed on sale with Messrs. Eyre and Spottiswoode, East Harding Street, E.C., at 1s. a copy net.

### Worcester General Infirmary.

The salary of the secretary of this infirmary has been increased from £125 to £150 to enable him to defray the cost of a girl to assist him a few days each week, to whom he pays 5s. a week. If the choice were given for hospital-secretaries throughout the country to choose between an assistant at 5s. a week or a war dietary for himself, which would he select?

### Annual Meetings.

Queen's Hospital for Children, Hackney Road, E.—In the Board Room of the hospital, on Wednesday, May 31, at 4 p.m., Colonel Lord William Cecil, C.V.O., in the chair.

National Sanatorium Association, Benenden, Kent.—At the offices of the National Union of Railwaymen, Unity House, Euston Road, N.W., Saturday, May 27, 5 p.m.

Miller General Hospital for South-East London, Greenwich Road, S.E.—At the hospital, Tuesday, June 6, at 5 p.m. Inspection of hospital at 4 p.m.

RATES OF SUBSCRIPTION: Twelve months, 6/6; Six months, 4/-; Three months, 2/-, post free to any address in the United Kingdom. Abroad, Twelve months, 8/8; Six months, 5/-; Three months, 2/6, post free.—Address The Subscription Dept., "THE HOSPITAL," The Hospital Building, 28/29 Southampton Street, Strand, London, W.C.

